# Simple graphical approach to investigate differences in transepithelial paracellular leak pathway permeability

**DOI:** 10.14814/phy2.15202

**Published:** 2022-03-11

**Authors:** Ashley Monaco, Josephine Axis, Kurt Amsler

**Affiliations:** ^1^ Department of Biomedical Sciences NYIT College of Osteopathic Medicine Old Westbury New York USA

**Keywords:** macromolecule, permeability, tight junction

## Abstract

Although many studies have reported differences in epithelial paracellular Leak Pathway permeability following genetic manipulations and treatment with various agents, the basis for these differences remains mostly unclear. Two primary mechanisms which could underlie differences in Leak Pathway permeability are differences in the density of Leak Pathway openings and differences in the opening size. Using a computational approach, we demonstrate that these two possibilities can be readily distinguished graphically by comparing the apparent paracellular permeabilities of a size panel of solutes measured across different cell layers. Using this approach, we demonstrated that depletion of ZO‐1 protein in MDCK Type II renal epithelial cells decreased Leak Pathway opening size and increased opening density. Depletion of ZO‐2 protein either had no effect or minimally decreased opening size and did not markedly change opening density. Comparison of MDCK Type II cells with MDCK Type I cells revealed that Type I cells exhibited a substantially smaller Leak Pathway permeability than did Type II cells. This lower permeability was due to a decrease in opening density with little or no change in opening size. These results demonstrate the utility of this approach to provide insights into the basis for observed differences in epithelial Leak Pathway permeability. This approach has wide applications including analysis of the molecular basis for Leak Pathway permeability, the effects of specific manipulations on Leak Pathway permeability properties, and the effects of permeation enhancers on Leak Pathway permeability properties.

## Graphical abstract

A simple graphical approach has been developed to investigate observed differences in epithelial paracellular Leak Pathway permeability. The approach distinguishes between permeability changes produced by differences in Leak Pathway opening size versus Leak Pathway opening density. This approach demonstrates that ZO‐1 depletion in MDCK II cells, which increases Leak Pathway permeability, decreases Leak Pathway opening size, and increases the opening density.

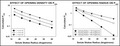

## 1 INTRODUCTION

The movement of large solutes across epithelial cell layers by movement through the tight junctions joining adjacent epithelial cells is mediated by the Leak Pathway (for reviews, see Monaco et al., 2021; Otani & Furuse, 2020; Shen et al., 2011). This pathway exhibits a relatively small capacity and is unaffected by the solute charge. Modulation of tight junction permeability has been implicated in both normal physiology and pathophysiology (see, e.g., Schleimer & Berdnikovs, 2017; Zuo et al., 2020). Studies have documented changes in Leak Pathway permeability produced by a wide range of stimuli including hydrogen peroxide (Basuroy et al., 2006; Janosevic et al., 2016), cytokines (Mullin et al., 1997; Van Itallie et al., 2010; Watson et al., 2005), mechanical stress (Samak et al., 2014), and additives used to enhance drug delivery (Brayden et al., 2015; Del Vecchio et al., 2012; McCarron et al., 2021). Manipulation of tight junction membrane protein content has also been reported to modulate epithelial cell Leak Pathway permeability (Al‐Sadi et al., 2011; Bilal et al., 2018; Tokuda et al., 2014; Van Itallie et al., 2009). The effects of the various stimuli are likely mediated via intracellular signaling pathways (Basuroy et al., 2006; Hasegawa et al., 1999; Janosevic et al., 2016; Jou et al., 1998; Sheth et al., 2009) and, possibly, effects on actomyosin function (Van Itallie et al., 2015; Bilal et al., 2018; He et al., 2020). Despite its importance, relatively little is known about the basis for differences in Leak Pathway permeability produced by various stimuli, manipulations, disease processes, and chemical agents.

Two major mechanisms to alter Leak Pathway permeability are changes in Leak Pathway opening density (the percentage of the surface area that is openings) and changes in Leak Pathway opening radius. The Renkin sieving equation (Renkin, 1954) has been employed as a mathematical model to determine some basic properties of the Leak Pathway (see, e.g., Buschmann et al., 2013; Kawedia et al., 2008). But, this approach has not been used extensively to determine the basis for observed changes in Leak Pathway permeability following experimental manipulations or when comparing different cell types. Inspection of the Renkin sieving equation suggests the behavior of Leak Pathway permeability as a function of solute size would be affected differentially by changes in opening radius versus changes in opening density. Knowledge of the basis for differences in Leak Pathway permeability has important implications for physiological and pathophysiological processes associated with changes in Leak Pathway permeability. It could also provide important insights for developing strategies to enable macromolecular therapeutics to cross the epithelial paracellular permeability barrier in a controlled manner.

To determine if differences in Leak Pathway permeability produced by differences in opening density versus opening radius can be distinguished based on their dependence on solute Stokes radius, we performed a theoretical computational analysis. This analysis confirmed that differences in opening density versus opening radius differentially affect Leak Pathway permeability dependence on solute Stokes radius in an easily distinguishable manner. This approach was then used to examine cases where differences in Leak Pathway permeability have been reported. Previous studies have reported that depletion of ZO‐1, a tight junction cytoplasmic protein, increased Leak Pathway permeability (Bilal et al., 2018; Tokuda et al., 2014; Van Itallie et al., 2009). The effect of ZO‐2 depletion on Leak Pathway permeability is unclear (Hernandez et al., 2007; Raya‐Sandino et al., 2017; Van Itallie et al., 2009). We asked if our approach could determine the basis for changes in Leak Pathway permeability caused by depletion of ZO‐1 versus ZO‐2 protein content in MDCK Type II renal epithelial cells. As a second test, we examined the basis for observed differences in Leak Pathway permeability in MDCK Type II cells versus MDCK Type I cells.

## 2 MATERIALS AND METHODS

### 2.1 Reagents

4 kDa fluorescein‐dextran, 10 kDa fluorescein‐dextran, 20 kDa fluorescein‐dextran, 40 kDa fluorescein‐dextran, and 70 kDa fluorescein‐dextran were obtained from Sigma‐Aldrich Chemicals. α‐Modification Minimal Essential Medium (αMEM) was obtained from Corning Cellgro. Heat‐inactivated fetal bovine serum was obtained from Atlanta Biologicals. Penicillin/Streptomycin Solution (100X) was obtained from MP Biomedicals. l‐Glutamine solution (200 mM) was obtained from Life Technologies. Trypsin/EDTA solution (0.25%) was obtained from HyClone. Antibodies used in these studies are as follows: rabbit anti‐ZO‐1 antibody (Invitrogen, catalog #40‐2200), rabbit anti‐ZO‐2 antibody (Life Technologies, catalog #711400), HRP‐conjugated goat anti‐rabbit F_(ab’)2_ fragment antibody (Jackson ImmunoResearch Laboratories, catalog #111‐036–003), and HRP‐conjugated goat anti‐rabbit F(_ab’)2_ fragment antibody (Invitrogen, catalog #31461).

### 2.2 Cell lines

Wild‐type MDCK Type II cell line was a kind gift from Dr. C.M. Van Itallie (NHLBI). ZO‐1 knockdown MDCK Type II cell line (ZO‐1 KD) and ZO‐2 knockdown MDCK Type II cell line (ZO‐2 KD) were kind gifts from Dr. A. Fanning (University of North Carolina). All knockdown MDCK Type II cell lines were obtained from the same parental MDCK Type II cell line. Characterization of the ZO‐1 KD cell line and the ZO‐2 KD cell line is described in Van Itallie et al. (2009). The MDCK I cell line was obtained from Sigma‐Aldrich. Measurement of transepithelial resistance using an EVOM (World Instruments) confirmed the low (MDCK II) versus high (MDCK I) resistance phenotypes. We did not independently authenticate the cell lines used in these studies.

### 2.3 Cell culture

Cell populations were grown as stock cultures maintained at a subconfluent density in tissue culture‐treated flasks in Complete Medium (αMEM supplemented with 10% fetal bovine serum plus 2‐mM L‐glutamine plus penicillin/streptomycin) at 37°C in a humidified 5% CO_2_ atmosphere. Cells were passaged every 3–4 days by detaching cells with trypsin/EDTA solution and replating at a 1:10–1:20 dilution onto tissue culture‐treated flasks. For flux experiments, detached cells were seeded onto permeable membrane filters (BD Biosciences; 25‐mm diameter, 0.4‐μm pore diameter) in 6‐well tissue culture plates containing 2‐ml Complete Medium in both the upper and lower compartments. The medium was replenished every 2–3 days. Twelve to thirteen days after seeding, the medium was replenished with serum‐free αMEM supplemented with 2‐mM L‐glutamine plus penicillin/streptomycin. Cell populations were incubated overnight and then used for flux assays as described previously (Caswell et al., 2013).

### 2.4 Paracellular permeability assay

The paracellular permeability of MDCK cell populations to large solutes (Leak Pathway) was determined by measuring the transepithelial movement of a size series of fluorescein‐dextrans (4, 10, 20, 40, and 70 kDa) at a final concentration of 80 μM as described previously (Caswell et al., 2013). Samples were collected periodically over a 2‐h time period. Flux assay data points are presented as mean ± standard deviation of triplicate independent samples. The presented flux curves are representative curves of 8–10 independent experiments. Apparent permeability (P_app_) is defined as (dQ/dt)/AC_o_ as described by Van Itallie et al. (2008). P_app_ values for each cell line are calculated from the average flux rates for each flux curve for each fluorescein‐dextran species. Presented P_app_ values for each cell line for each fluorescein‐dextran species represent the mean ± standard deviation of 8–10 independent experiments. The values for the kinematic viscosity (ν) of the fluorescein‐dextran solutions used in the experimental flux measurements were determined using an Ubbelohde‐type viscometer according to the manufacturer's instructions. Fluorescein‐dextran Stokes radii (r) were taken directly from the information provided by the supplier (Sigma‐Aldrich). The values for ν and r used in the calculations are shown in Table 1.

**TABLE 1:** Fluorescein‐dextran Stokes radii and solution kinematic viscosities

Fluorescein‐dextran size	Stokes radius (Å)	Kinematic viscosity [(Å^2^/sec) × 10^14^]
4 kDa	14	0.9614
10 kDa	23	0.9835
20 kDa	33	0.986
40 kDa	45	1.038
70 kDa	60	1.1168

Note

Fluorescein‐dextran Stokes radii are from the supplied data sheets (Sigma‐Aldrich). The kinematic viscosities of the fluorescein‐dextran solutions (80 μM in Ca‐Mg‐PBS) were measured using an Ubbelohde‐type viscometer (Cannon Instrument Company) according to the manufacturer's instructions.

### 2.5 Western blot analysis

Cell lysates were prepared from cell populations maintained under the conditions used for the measurement of paracellular permeability. Total cell protein lysates were prepared as previously described (Caswell et al., 2013). Western blotting was performed as previously described (Caswell et al., 2013). Primary antibody dilutions for western blotting were as follows: ZO‐1—1:500–1:2,000; ZO‐2—1:500–1:2,000. HRP‐conjugated anti‐rabbit F_c_ fragment antibodies were used at a dilution of 1:10,000–1:20,000. Signals were developed using the Pierce West Pico SuperSignal Reagent. Chemiluminescence signals were imaged using an Amersham Imager 600RGB. Presented blots are representative of at least four separate blots obtained from at least four independent sets of samples.

## 3 RESULTS

### 3.1 Computational modeling

We reasoned that the relationship between Leak Pathway apparent permeability (P_app_) and solute Stokes radius would be differentially affected by differences in opening density versus opening radius. To confirm this hypothesis, we carried out a computational modeling of the permeability of solutes of varying radii across a cell population with Leak Pathway openings of different radii versus different opening densities. For these calculations, we used the Renkin sieving equation (Renkin, 1954) to calculate P_app_ values for each solute species under each condition. The Renkin sieving equation has been used previously in multiple studies to computationally model paracellular permeability (Buschmann et al., 2013; Kawedia et al., 2008).

$$\begin{array}{c} P_{\text{app}}=\;{\left(\left(\varepsilon /\delta \right)\left(k_{B}T/6\pi \rho \nu r\right)(1‐r/\text{R)}\right)}^{2}\\ \left(1‐2.104\left(r/R\right)+2.09{\left(r/R\right)}^{3}‐0.95{\left(r/R\right)}^{5}\right) \end{array}$$

*ɛ* = opening density (total opening area/total surface area). *δ* = channel length = 2000 Å. k_B_ = Boltzmann constant = 1.380649 Å^2^g/^o^Ks^2^. T = temperature = 310^o^K. *ρ* = solution density = 10^−24^ g/Å^3^. ν = kinematic viscosity. r = solute Stokes radius. R = opening radius.

**FIGURE 1:**
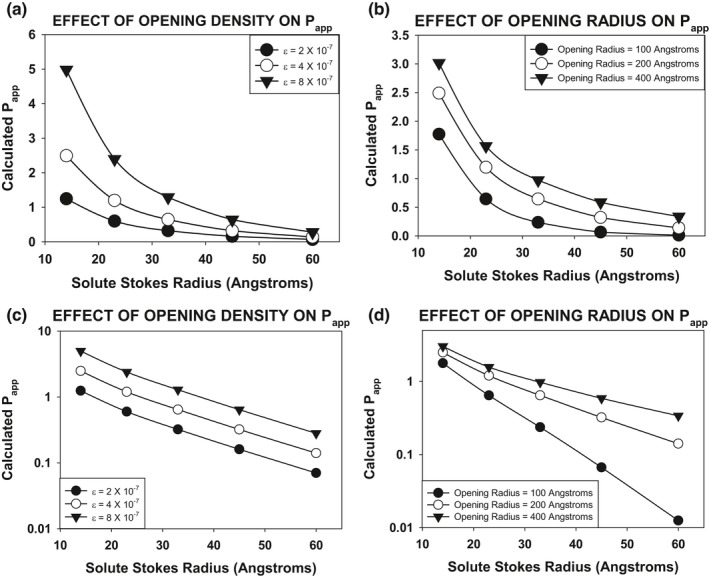
Computational analysis of the effect of varying opening radius versus opening density on P_app_ values as a function of solute Stokes radius. P_app_ values were calculated using the Renkin sieving equation (Renkin, 1954) with values as described in the text. When varying opening radius, the opening density was held constant at 4 × 10^−7^ (the percent of the total surface area that is openings). When varying opening density, the opening radius was held constant at 200 Å. The panels shown in (a) present the P_app_ data on a linear scale. The panels shown in (b) present the P_app_ data on a log scale

To determine the effect of varying opening density (*ɛ*), we used values of 2 × 10^−7^, 4 × 10^−7^, and 8 × 10^−7^. For these calculations, the opening radius (R) was held constant at 200 Å. To determine the effect of varying opening radius (R), we used values of 100 Å, 200 Å, and 400 Å. For these calculations, the opening density (*ɛ*) was held constant at 4 × 10^−7^. The results from these calculations are shown in Figure 1. Figure 1a shows the results of the calculations plotted using a linear y axis (P_app_). These calculations demonstrate a clear difference in the behavior of P_app_ versus solute radius when varying opening density versus opening radius. This difference in behavior is even more apparent when the results are displayed on a semilog plot (Figure 1b). Differences in opening radius (right‐hand panel) result in lines which diverge as a function of solute Stokes radius. The smaller the opening radius, the more rapidly the curve falls off as a function of solute Stokes radius. In contrast, differences in opening density (left‐hand panel) result in “parallel curves” on the semilog plot.

### 3.2 Effect of knockdown of ZO‐1 versus ZO‐2 on P_app_ as a function of solute Stokes radius

**FIGURE 2:**
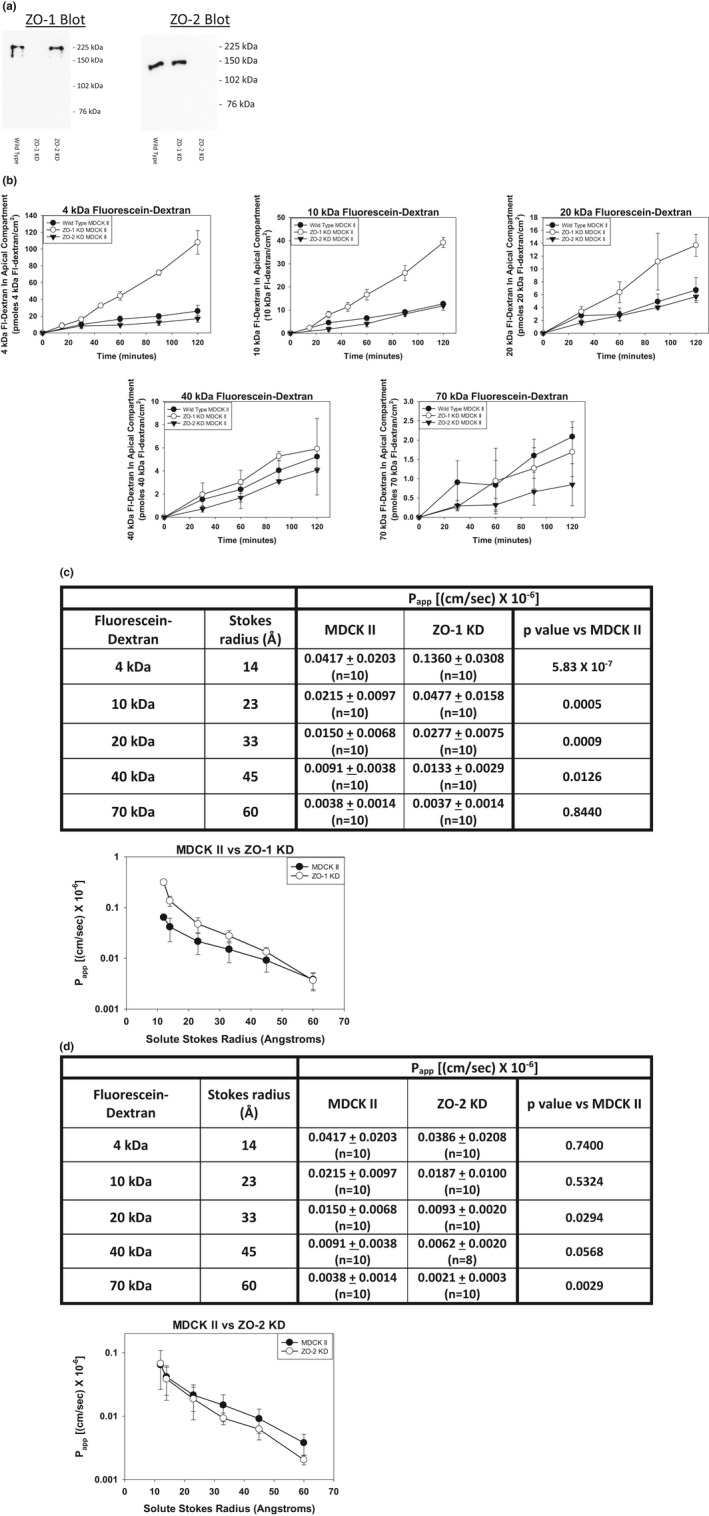
Comparison of fluorescein‐dextran flux versus time and P_app_ values versus solute Stokes radius in MDCK II cell monolayers, ZO‐1 KD cell monolayers, and ZO‐2 KD cell monolayers. (a) Knockdown of the appropriate protein, ZO‐1 or ZO‐2, is demonstrated by western blot. (b) Solute flux versus time for the size panel of fluorescein‐dextrans (4 kDa–70 kDa) for MDCK II cell monolayers, ZO‐1 KD cell monolayers, and ZO‐2 KD cell monolayers. Data are presented as mean ± standard deviation of triplicate independent samples. The flux curves are representative examples of 8–10 separate experiments. (c) P_app_ values as a function of solute Stokes radius for MDCK II versus ZO‐1 KD cell monolayers. Significance when comparing the P_app_ values for MDCK II versus ZO‐1 KD cell monolayers, determined by two‐tailed t‐test, are presented in the table. Curves for the P_app_ values versus solute Stokes radii are presented on the semilog plot. P_app_ values are the mean ± standard deviation of 8–10 independent experimental determinations of P_app_ for each fluorescein‐dextran species in each cell line. (d) P_app_ values as a function of solute Stokes radius for MDCK II versus ZO‐2 KD cell monolayers. Significance when comparing the P_app_ values for MDCK II versus ZO‐2 KD cell monolayers, determined by two‐tailed t‐test, are presented in the table. Curves for the P_app_ values versus solute Stokes radii are presented on the semilog plot. P_app_ values are the mean ± standard deviation of 8–10 independent experimental determinations of P_app_ for each fluorescein‐dextran species in each cell line

These computational results indicate that it may be possible to distinguish between changes in P_app_ produced primarily by changes in Leak Pathway opening density and those produced primarily by changes in Leak Pathway opening radius. As the first test of this approach, we compared wild‐type MDCK Type II renal epithelial cells (MDCK II) with ZO‐1 knockdown MDCK II cells (ZO‐1 KD). It has previously been reported that knockdown of the tight junction protein, ZO‐1, in MDCK II cells increases Leak Pathway permeability (Bilal et al., 2018; Tokuda et al., 2014; Van Itallie et al., 2009). To pursue this observation, we examined the flux of fluorescein‐dextrans of different sizes (4 kDa ‐ ~14 Å to 70 kDa ‐ ~60 Å) across monolayers of MDCK II cells and ZO‐1 KD cells. We also measured the fluxes of the fluorescein‐dextran size panel across monolayers of ZO‐2 knockdown MDCK II (ZO‐2 KD) cells. The effect of ZO‐2 knockdown on Leak Pathway permeability is unclear (Hernandez et al., 2007; Raya‐Sandino et al., 2017; Van Itallie et al., 2009). A western blot analysis of cell lysates from MDCK II cells, ZO‐1 KD cells, and ZO‐2 KD cells confirmed the targeted knockdown of ZO‐1 and ZO‐2 in the appropriate cell line (Figure 2a).

Representative flux curves for each fluorescein‐dextran across the three cell lines are shown in Figure 2b. The movement of each fluorescein‐dextran across monolayers of all three cell lines appeared linear with time over the 2‐h assay period. For the smaller fluorescein‐dextrans, 4 kDa through 20 kDa, the flux rate across the ZO‐1 KD cell monolayers was markedly faster than the movement of these fluorescein‐dextrans across monolayers of the MDCK II cells. As the fluorescein‐dextran Stokes radius increased, the difference between the flux rates for the ZO‐1 KD cells and the MDCK II cells diminished. The flux rates for each of the fluorescein‐dextrans appeared similar across the ZO‐2 KD cell line and the MDCK II cell line.

Apparent permeability (P_app_) values for each fluorescein‐dextran across each cell line were calculated from the measured flux rates. The values from multiple separate experiments for each cell line and fluorescein‐dextran were averaged. The average P_app_ values for the ZO‐1 KD cell monolayers were significantly greater than those for the MDCK II cell monolayers for all fluorescein‐dextrans except the 70 kDa fluorescein‐dextran (Figure 2c). The ZO‐2 KD cell monolayer average P_app_ values were not statistically different from the MDCK II cell monolayer values for the smaller fluorescein‐dextrans (4 kDa, and 10 kDa) but did exhibit significantly smaller average P_app_ values for the 20 kDa and 70 kDa fluorescein‐dextrans (Figure 2d). The average P_app_ value of the ZO‐2 KD cell monolayers for the 40 kDa fluorescein‐dextran was smaller than the average P_app_ value for the MDCK II cell monolayers but did not reach statistical significance at the *p *< 0.05 level.

Average P_app_ values were plotted as a function of solute Stokes radius on a semilog plot. The graph shown in Figure 2c compares the effect of solute Stokes radius on the average P_app_ values in the MDCK II cell line versus the ZO‐1 KD cell line. As expected, the average P_app_ values for both cell lines decreased with increasing solute Stokes radius. The average P_app_ values of the smaller fluorescein‐dextrans for the ZO‐1 KD cell line were statistically greater than the average P_app_ values for the MDCK II cell line. The ZO‐1 KD cell line average P_app_ values decreased more rapidly than did the MDCK II average P_app_ values as a function of solute Stokes radius such that, for the 70 kDa fluorescein‐dextran, there was no statistical difference between the two cell lines. The graph shown in Figure 2d compares the average P_app_ values for the MDCK II cell line versus the ZO‐2 KD cell line. The P_app_ values for the MDCK II cell monolayers and the ZO‐2 KD cell monolayers were similar, although there may be a slight divergence as solute Stokes radius increased.

### 3.3 Comparison of P_app_ as a function of solute stokes radius in MDCK type II cell line versus MDCK type I cell line

**FIGURE 3:**
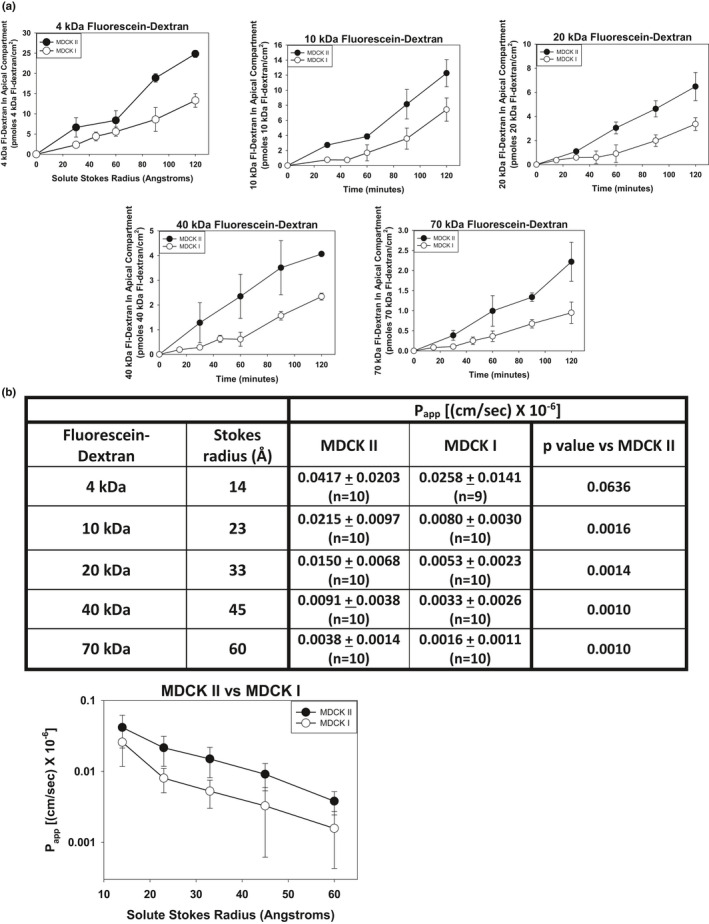
Comparison of fluorescein‐dextran flux versus time and P_app_ values versus solute Stokes radius in MDCK II cell monolayers and MDCK I cell monolayers. (a) Solute flux versus time for the size panel of fluorescein‐dextrans (4 kDa–70 kDa) for MDCK II cell monolayers and MDCK I cell monolayers. Data are presented as mean ± standard deviation of triplicate independent samples. The flux curves are representative examples of 8–10 separate experiments. (b) P_app_ values as a function of solute Stokes radius for MDCK II versus MDCK I cell monolayers. Significance when comparing the P_app_ values for MDCK II versus MDCK I cell monolayers, determined by two‐tailed t‐test, are presented in the table. Curves for the P_app_ values versus solute Stokes radii are presented on the semilog plot. P_app_ values are the mean ± standard deviation of 8–10 independent experimental determinations of P_app_ for each fluorescein‐dextran species in each cell line

We then compared the effect of solute Stokes radius on flux rate and average P_app_ values in the MDCK II cell line versus the MDCK Type I (MDCK I) cell line. Previous studies have demonstrated that MDCK I cell monolayers exhibit a markedly lower Pore Pathway permeability (Richardson et al., 1981; Stevenson et al., 1988). It is unclear if they also exhibit a lower Leak Pathway permeability. The fluxes of the fluorescein‐dextran size panel across monolayers of MDCK II cells and MDCK I cells are shown in Figure 3a. MDCK I cell monolayers exhibited slower permeability than did MDCK II cell monolayers for all fluorescein‐dextrans examined. Average P_app_ values were calculated for the MDCK I cell line as described above. Compared to MDCK II cell monolayers, monolayers of MDCK I cells exhibited statistically slower average P_app_ values for each of the fluorescein‐dextrans examined except the 4 kDa fluorescein‐dextran (Figure 3b). While the average 4 kDa fluorescein‐dextran P_app_ value was slower in the MDCK I versus the MDCK II cell line, the difference did not reach statistical significance at the *p *< 0.05 level. The P_app_ values for the MDCK I cells and MDCK II cells were plotted on a semilog plot (Figure 3b). The curve for the average P_app_ values for the MDCK I cell line as a function of solute Stokes radius appeared to parallel the curve for the MDCK II P_app_ values.

## 4 DISCUSSION

We have used an analysis of the effect of solute Stokes radius on P_app_ values to assess the relative importance of opening density versus opening radius in producing observed differences in Leak Pathway flux rates. We first performed a theoretical computational experiment to investigate how the effect of changing either of these parameters would affect P_app_ values as a function of solute Stokes radius. The results of this computational analysis indicated that changes primarily to the Leak Pathway opening density would manifest as “parallel” curves when plotting P_app_ values versus solute Stokes radii on a semilog plot. Changes primarily to the Leak Pathway opening radius would manifest as curves with diverging “slopes” on the semilog plot.

In addition to identifying samples with differences in Leak Pathway opening sizes, this approach has the potential to provide a measure of the relative difference in Leak Pathway opening density. For samples exhibiting similar opening sizes but different P_app_ values, i.e., “parallel” curves on the semilog plot, the ratio of the solute P_app_ values for each sample will provide a relative difference in the Leak Pathway opening densities. For samples which exhibit different Leak Pathway opening radii, i.e., the curves which diverge on the semilog plot, the ratio of the P_app_ values of the smallest solute will provide a minimum relative difference in opening density. The P_app_ values for the smallest solute should be used because this solute will experience the least hindrance from the opening walls. Measurement of the flux of a smaller solute in the two samples could provide a more accurate measure of the relative difference in Leak Pathway opening density.

One limitation of this analysis is the requirement that the solute Stokes radii be sufficiently large to interact with the Leak Pathway openings. If all of the solute Stokes radii are very much smaller than the Leak Pathway opening radii, the openings will not hinder the passage of solutes through the opening. In this circumstance, the flux for each solute will approach the rate of free diffusion for that solute, limited only by the proportion of the total surface area that is openings.

A second limitation of this analysis is that differences in either opening radius and/or opening density between samples must be sufficiently large to reveal clear differences in this graphical approach. Minimal changes in either parameter would likely yield samples with similar flux rates and similar P_app_ values. As observed here with the wild‐type MDCK II cell monolayers versus the ZO‐2 KD cell monolayers, such a result cannot distinguish between no effect and having a minor effect on one or more opening parameters.

Previous studies have used the Renkin sieving equation to compare samples exhibiting different Leak Pathway permeabilities (Buschmann et al., 2013; Cavanaugh et al., 2006; Durbin et al., 1956; Kawedia et al., 2008). There are, however, multiple mathematical formulations to analyze paracellular permeability (see, e.g., Dechadilok & Deen, 2006; Renkin, 1954). These mathematical formulations have the same basic form. The free diffusion equation is modified by a factor that takes into account the opening density and opening length. This factor is not dependent on the opening radius or solute radius. The free diffusion equation is further modified by two factors, a hindrance factor (interaction of the solute with the opening surface) and a frictional factor (interaction of the solute with the walls as it traverses the passage). Both of these factors are dependent on both opening radius and solute radius. Since these relationships hold regardless of the specific mathematical formulation, it is possible to separate the effects of opening density, which are insensitive to solute size, from opening radius, which are affected by solute size. Our graphical approach obviates the need for extensive calculations and allows a simple comparison of two samples via direct permeability measurements over a range of solute sizes.

To assess the utility of this approach, we examined two cases in which differences in Leak Pathway paracellular permeability have been reported. Multiple studies have reported that knockdown of ZO‐1 protein in the MDCK II cell line increases Leak Pathway permeability (Bilal et al., 2018; Tokuda et al., 2014; Van Itallie et al., 2009). Our results indicate that the difference between the parent cell line and the knockdown cell line diminishes with increasing solute Stokes radius such that there was no difference in average P_app_ values for the 70 kDa fluorescein‐dextran. Plotting the average P_app_ values as a function of solute Stokes radius on a semilog plot revealed that the curve for the ZO‐1 KD cell line values declined more rapidly than the curve for the MDCK II cell line values. Based on our computational analysis, this suggests that the ZO‐1 KD cell monolayers possess Leak Pathway openings with a smaller radius than do MDCK II cell monolayers. The fact that the ZO‐1 KD cell monolayers exhibit substantially higher P_app_ values for the smaller fluorescein‐dextrans, combined with the smaller opening radius, indicates the ZO‐1 KD cell monolayers possess a greater density of these smaller Leak Pathway openings. Based on the discussion above, the ZO‐1 KD cell monolayers express at least a 3‐fold greater number of Leak Pathway openings compared to MDCK II cell monolayers.

It has been suggested that Leak Pathway openings are sites of transient breaks in the tight junction strands, possibly due to altered tension at the site (Anderson et al., 2004; Arnold et al., 2017; Charras & Yap, 2018; Hatte et al., 2018; Stephenson et al., 2019). ZO‐1, either as homodimers or as heterodimers with ZO‐2, crosslinks the tight junction membrane proteins and links them to the actin cytoskeleton (Rodgers et al., 2013; Van Itallie et al., 2009). ZO‐1 KD cells exhibit altered apical actin organization (Odenwald et al., 2018; Van Itallie et al., 2009) and abnormal effects of manipulation of actomyosin contractility on Leak Pathway permeability (Bilal et al., 2018; Van Itallie et al., 2009). ZO‐1 is under actomyosin‐mediated tension in MDCK II cells (Haas et al., 2020). The F‐BAR protein, TOCA‐1, organizes branching actin networks (Liu et al., 2015). ZO‐1 binds TOCA‐1 and targets it to the tight junction region (Van Itallie et al., 2015). Knockout of TOCA‐1 in MDCK II cells both decreased tension on the tight junction (Van Itallie et al., 2015) and increased Leak Pathway permeability (Bilal et al., 2018; Van Itallie et al., 2015). Yu et al. (2010) demonstrated that disruption of actomyosin function by several compounds stabilized ZO‐1 at the tight junction region and increased transepithelial resistance. ZO‐1 protein expression, however, did not decrease the frequency of strand breaks and reannealing in a fibroblast model system transfected with claudin‐2 (Van Itallie et al., 2017). A recent study reported that occludin and tricellulin contributed to the complexity of the claudin strands (Saito et al., 2021). Decreased strand complexity was correlated with increased Leak Pathway permeability. Since ZO‐1 mediates crosslinking among the tight junction membrane proteins, ZO‐1 depletion and its associated alterations in apical actin organization and tight junction tension may affect strand integrity and/or complexity leading to increased Leak Pathway permeability. This possibility requires further investigation.

P_app_ values for the ZO‐2 KD cell line declined slightly more rapidly than P_app_ values for the MDCK II cell line when plotted on the semilog plot. This suggests that ZO‐2 depletion may produce a slight decrease in Leak Pathway opening size, although this remains unclear. Our results are consistent with the results of both Van Itallie et al. (2009) and Raya‐Sandino et al. (2017). P_app_ values for smaller fluorescein‐dextrans were not statistically different (Van Itallie et al., 2009), but a statistically significant difference was observed for larger fluorescein‐dextrans (Raya‐Sandino et al., 2017). In contrast to the effect of ZO‐1 depletion, ZO‐2 depletion does not appear to increase opening density. These results suggest that ZO‐1 has a much greater role than ZO‐2 in regulating Leak Pathway permeability. If Leak Pathway permeability does represent transient breaks in the claudin strands, these results would suggest that ZO‐1 is more important for maintaining strand integrity.

The fluorescein‐dextran flux data demonstrate that MDCK I cell monolayers exhibit a lower Leak Pathway permeability compared to MDCK II cell monolayers, in addition to their lower Pore Pathway permeability (Richardson et al., 1981; Stevenson et al., 1988). The P_app_ versus solute Stokes radius curve generated for the MDCK I cell line appeared, for the most part, to “parallel” the curve for the MDCK II cell line. This argues against a major difference in Leak Pathway opening radius in this cell line compared to the MDCK II cell line. The results suggest that the MDCK I cell monolayers possess a lower Leak Pathway opening density compared to the MDCK II cell monolayers, but the Leak Pathway openings are of a similar radius. Based on the P_app_ values, the MDCK I cell monolayers express on the order or a third to a half the density of Leak Pathway openings expressed by the MDCK II cell monolayers.

MDCK I cells express many of the tight junction proteins expressed by MDCK II cells (see, e.g., Lipschutz et al., 2005). Multiple differences have been observed, however, in tight junction protein content (see, e.g., Furuse et al., 2001). MDCK I cells express higher levels of claudin‐1, claudin‐4, and occludin. MDCK I and MDCK II cells express similar levels of ZO‐1. MDCK II cells express substantial levels of claudin‐2, whereas MDCK I cells are deficient in claudin‐2 (Furuse et al., 2001). It has been suggested that claudin‐2 may alter tight junction strand stability, thereby increasing Leak Pathway paracellular permeability (Luettig et al., 2015), but this has been disputed in a recent study (Raju et al., 2020). In addition, the introduction of claudin‐2 into MDCK I cells did not alter the permeability to either 4 kDa or 40 kDa fluorescein‐dextran, although it did decrease transepithelial resistance (increase cation permeability) markedly (Furuse et al., 2001). The decrease in transepithelial resistance with claudin‐2 expression is consistent with claudin‐2 being a cation pore‐forming claudin (Amasheh et al., 2002). The results, however, do not support a role for claudin‐2 in mediating the difference in Leak Pathway permeability observed between MDCK I and MDCK II cell monolayers.

As discussed above, a current hypothesis states that Leak Pathway openings are transient breaks in the tight junction strands (see, e.g., Anderson et al., 2004; Tervonen et al., 2019; Watson et al., 2001; Zihni et al., 2016). It is, therefore, unexpected that the Leak Pathway opening sizes in two different epithelial cell lines (MDCK II and MDCK I), which exhibit different Leak Pathway permeabilities, are similar. This raises the possibility that Leak Pathway opening size might be a consistent property across epithelia. Previous studies have reported similar Leak Pathway opening sizes in rabbit gallbladder epithelia (Van Os et al., 1973), frog gastric mucosa (Durbin et al., 1956), bullfrog alveolar epithelia (Kim & Crandall, 1983), rat alveolar Type II epithelia (Cavanaugh et al., 2006), rat submandibular gland epithelial cells (Kawedia et al., 2008), and Caco‐2 human intestinal epithelial cells (Buschmann et al., 2013). This possibility needs to be investigated further since it would have significant implications for Leak Pathway opening architecture.

In summary, we have demonstrated the ability of a computational approach to distinguish between the effects of Leak Pathway opening density versus opening radius in epithelial cell lines and tissues exhibiting different Leak Pathway permeabilities. Our results demonstrated that ZO‐1 depletion in MDCK II cells produced both a decrease in Leak Pathway opening radius and an increase in Leak Pathway opening density. Depletion of ZO‐2 had little or no effect on Leak Pathway opening radius and no effect on opening density. The comparison of the MDCK II and MDCK I cell lines revealed that the lower Leak Pathway permeability exhibited by the MDCK I cell line was due primarily to a decrease in opening density with no obvious change in opening radius. These studies demonstrate the potential of this approach to elucidate how specific tight junction protein manipulations affect these Leak Pathway parameters. In addition, it can be applied to the analysis of the basis for changes in Leak Pathway permeability produced by various physiologic/pathophysiologic compounds, as well as compounds designed to modulate Leak Pathway permeability to enhance the delivery of macromolecule drugs. This relatively simple graphical approach has the potential to provide important new insights into Leak Pathway parameters that, to date, have not been readily accessible to analysis.

## CONFLICT OF INTEREST

The authors do not have any conflict of interest.

## AUTHOR CONTRIBUTIONS

AM – Experimental design, experimentation, manuscript writing, manuscript review. JA – Experimental design, experimentation, manuscript writing, manuscript review. KA ‐ Experimental design, experimentation, manuscript writing, manuscript review.
